# Large Scale Screening of *Epichloë* Endophytes Infecting *Schedonorus pratensis* and Other Forage Grasses Reveals a Relation Between Microsatellite-Based Haplotypes and Loline Alkaloid Levels

**DOI:** 10.3389/fpls.2019.00765

**Published:** 2019-06-12

**Authors:** Giovanni Cagnano, Niels Roulund, Christian Sig Jensen, Flavia Pilar Forte, Torben Asp, Adrian Leuchtmann

**Affiliations:** ^1^DLF Trifolium A/S, Roskilde, Denmark; ^2^Department of Molecular Biology and Genetics, Faculty of Science and Technology, Research Centre Flakkebjerg, Aarhus University, Slagelse, Denmark; ^3^Institute of Integrative Biology, ETH Zurich, Zurich, Switzerland

**Keywords:** *Epichloë*, genetic diversity, grass endophytes, interspecific hybrids, microsatellite markers, *Schedonorus*, loline

## Abstract

Species belonging to the Festuca-Lolium complex are often naturally infected with endophytic fungi of genus *Epichloë*. Recent studies on endophytes have shown the beneficial roles of host-endophyte associations as protection against insect herbivores in agriculturally important grasses. However, large-scale screenings are crucial to identify animal friendly strains suitable for agricultural use. In this study we analyzed collected populations of meadow fescue (*Schedonorus pratensis*) from 135 different locations across Europe, 255 accessions from the United States Department of Agriculture and 96 accessions from The Nordic Genetic Resource Centre. The analysis also included representatives of *S. arundinaceus*, *S. giganteus*, and *Lolium perenne*. All plants were screened for the presence of *Epichloë* endophytes, resulting in a nursery of about 2500 infected plants from 176 different locations. Genetic diversity was investigated on 250 isolates using a microsatellite-based PCR fingerprinting assay at 7 loci, 5 of which were uncharacterized for these species. Phylogenetic and principal components analysis showed a strong interspecific genetic differentiation among isolates, and, with *E. uncinata* isolates, a small but significant correlation between genetic diversity and geographical effect (*r* = 0.227) was detected. Concentrations of loline alkaloids were measured in 218 infected meadow fescue plants. Average amount of total loline and the proportions of the single loline alkaloids differed significantly among endophyte haplotypes (*P* < 0.005). This study provides insight into endophyte genetic diversity and geographic variation in Europe and a reference database of allele sizes for fast discrimination of isolates. We also discuss the possibility of multiple hybridization events as a source of genetic and alkaloid variation observed in *E. uncinata*.

## Introduction

Many grasses of the subfamily Pooideae form symbiotic relationships with filamentous fungi of the Clavicipitaceae family belonging to the genus *Epichloë* (Schardl et al., 1997). Among grasses and endophytic fungi there is a continuum of symbiotic interactions that range from antagonistic to clearly mutualistic (Schardl and Clay, 1997; Schmid et al., 2017). *Epichloë* endophytes grow asymptomatically in the intercellular spaces of the aerial tissues of the host plants and, in most of the asexual species, they do not spread by infecting neighboring plants but they are exclusively seed-transmitted from previously infected hosts (Schardl, 1996). At seed maturity, the endophyte is found between the pericarp and the aleurone layer, and between the cells of the embryo/scutellum, an area called “the infection layer” (Johnson et al., 2013). In some species the efficiency of vertical transmission is close to 100% and usually all seeds are infected (Schardl, 1996). Asexual *Epichloë* species may arise from sexual species that lost the ability to sexually reproduce (e.g., *E. festucae* var. *lolii*) or from interspecific hybridizations between sexual and/or asexual *Epichloë* species (Craven, 2003). Hybridizations may occur within host plants that are co-infected by different strains through a process known as vegetative hyphal fusion (VHF) or anastomosis followed by nuclear fusion (Shoji et al., 2015). Interspecific hybrids have an allopolyploid-like genome which is the result of the combination of two or more parental chromosome sets. Several studies support the evidence of the prevalence of interspecific hybrids amongst *Epichloë* species (Moon et al., 2004; Ghimire et al., 2011): hybridization might reduce the effects of deleterious mutations that accumulate in clonal genomes, the so-called “Muller’s ratchet” (Muller, 1964), and provide the endophyte with an additional set of genes for alkaloid biosynthesis which will eventually improve host fitness, and through it, the endophyte fitness itself (Selosse and Schardl, 2007).

Presence of *Epichloë* endophyte was generally assumed to be undesirable in the late 70s, when they were identified as the causal agent of fescue toxicosis and ryegrass stagger (Bacon et al., 1977; Fletcher and Harvey, 1981; Gallagher et al., 1981). In order to preserve the health of livestock, endophyte free varieties of tall fescue and perennial ryegrass were released, but their yield and persistence were not comparable to the endophyte infected grasses especially in areas with strong environmental pressure, such as New Zealand (Latch and Christensen, 1982; Bouton et al., 1993). The increasing knowledge on *Epichloë* endophytes and their secondary metabolites led researchers to reconsider their use in agriculture. This led private and public research organizations to focus on large-scale screenings in order to isolate animal-friendly endophytes still harboring deterrent and insecticidal properties, opening a new market for artificially infected grass cultivars (Bouton et al., 2002; Johnson et al., 2013). The “ideal endophyte” to be exploited in a more sustainable agriculture would be an asexual *Epichloë* strain with high production of water soluble insecticide and nematocide alkaloids; low to no production of alkaloid toxic to livestock; high compatibility with different species of the Festuca-Lolium complex; a stable profile and amount of secondary metabolites when inoculated in non-native grasses; high persistence in top varieties through the generations. Particularly interesting, in this respect, are two species of endophytes isolated from meadow fescue (*Schedonorus pratensis* Huds.): *E. uncinata* (Gams et al., 1990) and *E. siegelii* (Craven et al., 2001). These species produce high levels of lolines, a group of aminopyrrolizidine derivatives with deterrent and insecticidal properties without side effects on livestock (Patchett, 2007; Schardl et al., 2007). Loline alkaloids occur in several forms, the most common and abundant ones are N-formylloline (NFL), N-acetylloline (NAL), N-acetylnorloline (NANL), and N-methylloline (NML). These alkaloids normally accumulate in the aerial parts of the plant but they can also be found, in variable amounts, in the roots: the amount stored in below ground tissues can increase according to the presence of insects feeding on the roots themselves (Patchett et al., 2008).

In this study we (i) determined the incidence of *Epichloë* endophytes in Pooideae grasses (primarily *S. pratensis* but also *S. giganteus, S. arundinaceus*, and *L. perenne*) collected at various sites in Europe; (ii) screened meadow fescue accessions labeled as wild or landraces from the United States Department of Agriculture (USDA) and Nordic Genetic Resource Centre (NordGen); (iii) identified isolates from *S. arundinaceus* var. *glaucescens* as FaTG-5 through a phylogenetic analysis of *tubB* and *tefA* sequences, along with morphological examinations and microsatellites fingerprinting; (iv) tested the descriptive and discrimination power of 5 microsatellite markers on *E. uncinata, E. coenophiala*, *E. siegelii*, *E. festucae*, and *E. festucae* var. *lolii*; (v) investigated the genetic diversity of these species; and (vi) measured the amount of loline alkaloids in the isolates and tested their associations with endophyte microsatellite profiles.

## Materials and Methods

### Plant Material

*Schedonorus pratensis*, *Schedonorus arundinaceus*, *Schedonorus Giganteus,* and *Lolium perenne* plants were collected during the summer 2016 and summer 2017 in Denmark, Sweden, Norway, Italy, Germany, and Austria. The 135 collection sites were meadows located near roadsides, in isolated environments, supposedly not contaminated by cultivated grasses. Whenever possible, approximately 15 to 20 plants were sampled from each location. Latitude, longitude, and altitude data of all collection sites were recorded. Plants were planted in pots (9 cm × 11 cm) with standard peat (En brown 06 W 30P, Kekkilä Group, Vantaa, Finland), watered regularly and grown in the greenhouse with 16 light hours at approximately 15–24°C.

A total of 255 *S. pratensis* accessions were requested from USDA and 92 from NordGen. Only accessions labeled as wild or semi-wild were chosen. Seeds were germinated in the same conditions as described above. A map and a detailed database of the plant material used in this study are provided in Supplementary Figure S1 and Supplementary Table S1.

### Endophyte Detection, Isolation, and Identification

The presence of *Epichloë* endophytes was determined in two tiller sections from each of the collected plants, 6 weeks after the transplant, using the immunoblot assay “Phytoscreen Field Tiller Endophyte Detection Kit” (Cat. #ENDO797-3; Agrinostics Ltd. Co.) described by Hiatt et al. (1999) according to manufacturer’s description. A PCR-based approach was used to screen the high number of genotypes from the genebank accessions (approximately 17,000). The presence of the endophyte was initially tested with an *in planta* assay using microsatellite markers B10 and B11 described by Moon et al. (1999) on 50 stems per accession, split in 5 bulk samples of 10 stems each. Accessions where no amplifications was detected were considered endophyte-free and discarded, whereas accessions that produced amplicons were moved in single plant trays, 50 plants per accession, and tested with the “Phytoscreen Field Tiller Endophyte Detection Kit.” The infection rate of every population was scored as the ratio of infected to total number of tested plants, descriptive statistics such as mean, confidence interval of 95% (95% CI), standard error (SE), and sample size (n) were calculated using Microsoft Excel (2016). High-resolution DNA flow cytometry was used to confirm the species of the infected populations according to Jensen et al. (2007).

Pure cultures of endophytes were obtained from pieces of surface-sterilized pseudostems on potato dextrose agar (PDA) as described elsewhere (An et al., 1993; Schardl and An, 1993). To study colony growth, 2 mm^2^ plugs of mycelium were placed at the center of PDA plates and grown in the dark at 24°C. Colony diameter was measured from 5 replicate plates after 14, 21, and 28 days. For microscopic examinations of conidia and conidiophores, agar plates were inoculated with a suspension obtained by macerating 2 mm^3^ of culture in 100 μL of sterile water and kept in a dark growth chamber at 24°C for 7 days until conidiation occurred. The examination was performed with an Olympus BH-2 microscope (Olympus Optical Co., Hamburg) and photographs taken with a Canon EOS 600D camera. Measurements of conidiogenous cells (length and width at tip and base) and conidia and conidiophores (length and width) were taken from 20 structures each in 3 different isolates using an ocular micrometer at 1000×.

### DNA Amplification, Sequencing and Analysis of *tefA*, and *tubB* Genes

DNA extraction, PCR reaction, and gene cloning were performed using the method described by Oberhofer and Leuchtmann (2012). The nuclear genes for β-tubulin (*tubB*) and translation elongation factor 1-alpha (*tefA*) were PCR-amplified from genomic DNA. Both genes have been widely used for phylogenetic analysis of broad taxonomic range of endophytes worldwide. The primers used for the PCR reaction were 5′-TGG TCA ACC AGC TCA GCA CC-3′ (forward) and 5′-TGG TCA ACC AGC TCA GCA CC-3′ (reverse) for *tubB*, and primers 5′-GGG TAA GGA CGA AAA GAC TCA-3′ (forward) and 5′-CGG CAG CGA TAA TCA GGA TAG-3′ (reverse) for *tefA* (Craven et al., 2001). Isolates of the accession PI347572 were cloned into bacterial plasmids for separating alleles into different *Escherichia coli* colonies. Correct product size was verified by gel electrophoresis. Copies of both genes were sequenced and manually edited with Sequencher 10.4.1 (Gene Codes Corporation, United States). Sequences were aligned in GENEIOUS version 6.1.3 (Bio-matters Ltd, Auckland, New Zealand) along with sequences from representative *Epichloë* species using default alignment parameters, gaps were removed, and ambiguously aligned sites were checked manually and adjusted if needed. Sequences were deposited in GenBank: *tef*A: MK423914 and MK423915; *tub*B: MK423916 and MK423917.

Maximum likelihood (ML) trees were constructed with MEGA X (Kumar et al., 2018) with default parameters and 1,000 bootstrap replicates. Gene sequences available in Genbank of endophyte species related to the ones analyzed in this study and relevant putative ancestors were included in the dataset, each tree is provided with corresponding Genbank numbers.

### Microsatellite Analysis

Genomic DNA was extracted from infected stems on a Quadra 96 Model 320 robotic system (Tomtec Inc., Hamden, CT, United States) using the method described previously by Brazauskas et al. (2011). Two hundred fifty samples were characterized for their allelic variation at 7 microsatellites loci: B10 and B11 published by Moon et al. (1999) and E08, E29, E33, E36, and E39 published by Schirrmann et al. (2014). PCRs were performed in 10 μl volumes containing 4 μl of genomic DNA, 2 mM MgCl_2_, 0.25 mM of each deoxynucleotide triphosphate (dATP, dCTP, dGTP, and dTTP), 4 μM of each primer (Eurofins genomics, Ebersberg, Germany), 0.4 U of Taq DNA polymerase with 1× key buffer (Mg^2+^ free) (Cat. No. 733-1313, VWR International, Leuven, Belgium). Each primer pair was fluorescently labeled either with DY-682 or with DY-782 (Eurofins genomics, Ebersberg, Germany). Reactions were carried out in a Mastercycler gradient 5331 (Eppendorf AG, Hamburg, Germany) programmed with 2 min of initial denaturation at 95°C followed by 34 cycles of 95°C for 20 s, 56°C for 30 s, and 72°C for 1 min, with a final extension of 72°C for 10 min.

Lab internal standards (*E. uncinata*, *E. siegelii*, *E. festucae* var. *lolii*, *E. coenophiala*, FaTG-2, endophyte free plant and negative control) were used in each PCR reaction. The products were then electrophoresed using a LI-COR model 4200 automated fluorescent DNA sequencer (Middendorf et al., 1992) (LI-COR, Lincoln, NE, United States). Gel dimensions were 25 cm long and 0.25 mm thick. The gel contained 7 M urea and 7.0% SequaGel XR concentrate (National Diagnostics, Atlanta, Georgia). The running buffer was 0.4X TBE. The gel was run at 2000 V constant voltage, and the gel temperature was maintained at 50°C. The size of the amplicons was estimated by comparison to a size ladder with 42, 44, 125, 126, 150, 151, 193, 251, 280, 327, 328, 414, and 551 base pair fragments markers. The accuracy of the size estimates is specific to the electrophoretic separation conditions and a confidence interval of ±5 bp should be considered.

### Loline Alkaloid Analysis

In March 2018, 218 endophyte infected *S. pratensis* plants were trimmed, moved in bigger pots (35 cm × 30 cm) with standard peat (En brown 06 W 30P, Kekkilä Group, Vantaa, Finland), watered regularly and grown in the same greenhouse room with 16 light hours at approximately 15–24°C. In June 2018 samples from the basal part of the tiller were harvested and freeze-dried in the same day and subsequently powdered in a laboratory mill. About 50 mg of each sample were sent in duplicate to AgResearch Grasslands (Palmerston North, New Zealand), were the analysis was performed using a gas chromatographic method described by Baldauf et al. (2011).

### Data Analysis

Microsatellite data were coded with a tetraploid-like format so that *E. coenophiala* samples, which present three alleles in 5 out of 7 microsatellites, could be included in the analysis. Null-alleles were regarded as missing values. Number of multi-locus genotypes (MLG), allelic richness, Simpson’s (1949) Index, evenness (Grünwald et al., 2003), Shannon-Wiener Index of MLG diversity (Shannon, 1948), Stoddart and Taylor’s (1988) Index of MLG diversity were determined using the *poppr* package (Kamvar et al., 2014) in R (R version 3.4.2). The Simpson’s Index was corrected for sample size multiplying it by n/(n–1) as well as the Stoddart and Taylor’s diversity index, which was scaled by sample size and expressed as percentage. The software GenAlex v. 6.5 (Peakall and Smouse, 2012) was used to determine the Nei’s (1978) unbiased genetic identity and diversity, the number of alleles, the number of effective alleles and the Shannon’s information index (Sherwin et al., 2006) at each locus. Assessment of genetic relatedness between MLGs was performed using the function *provesti.dist* which calculates Provesti’s genetic distance. The function *aboot* runs a bootstrap analysis set up on 10,000 bootstrap replicates, treating loci as independent units, it was visualized with a dendrogram created using the unweighted pair-group method with arithmetic average (UPGMA). Principle component analysis (PCA) was performed to complement model-based clustering methods and to test the ability to distinguish haplotypes. Matrixes comparison using Mantel test was performed between the genetic similarity matrix with the cophenetic matrix. The change in genetic similarity associated with increasing spatial distance between individuals was investigated by testing and plotting spatial autocorrelation at above distance intervals. A geographical distance matrix of Euclidean distances (km) was computed between all pairwise combinations sites from their GPS coordinates with the R package *geosphere* (Hijmans et al., 2017) and tested against a genetic similarity matrix. The R function *mantel.correlog* in the package *vegan* (Oksanen et al., 2018) was used to compute multivariate Mantel correlogram using Pearson correlation, the Sturge equation to estimate distance classes, the Bonferroni progressive correction (α = 0.05) and 9999 permutations for significance tests. The concentrations of loline alkaloids were compared between haplotypes using the Kruskal-Wallis test by rank, a non-parametric alternative to one-way ANOVA test which could not be used because its assumptions were not met. The output of the function *kruskal.test* tells if the concentration of loline alkaloids were significantly different between haplotypes, but the Wilcoxon rank sum test (function *pairwise.wilcox.test*) was needed to calculate pairwise comparisons between haplotypes.

## Results

### Infection Rates of Grass Populations and Accessions

A total of 2008 plants (1764 *S. pratensis*, 42 *S. giganteus*, 63 *S. arundinaceus*, and 139 *L. perenne*) were collected from 135 different locations at different altitudes (from 0 to 1740 m) and longitudes and screened for the presence of *Epichloë* endophytes with a tissue-print immunoblot assay. Infection rates (IRs), calculated for each location as the ratio of infected (E+) plants, and the number of analyzed plants, showed a large variation among the sites, spanning from 0 to 100% (Table 1). Sweden was the country where wild and semi-wild habitats containing meadow fescue were easiest to find and infected plants with IRs from 24 to 100% (mean = 87.6%; 95% CI ± 5.1%; *SE* = 2.5%; *n* = 42) were found in 42 of 42 sites. Also in Norway all the collection sites were found to host infected plants with high IRs, from 68 to 100% (mean = 90.6%; 95% CI ± 8.3%; *SE* = 3.6%; *n* = 9), but the occurrence of meadow fescue was lower and it was difficult to find isolated habitats due to the higher frequency of farms and grass cultivated fields in the Østfold area close to the border with Sweden. The average IR was slightly lower in the collections made in the Alpine regions, across the Italian and Austrian border where it ranged from 6.7 to 100% (mean = 76.3%; 95% CI ± 10.3%; *SE* = 5%; *n* = 24) and 25 to 95% (mean = 69.3%; 95% CI ± 14.7%; *SE* = 6.7%; *n* = 12), respectively. Regarding the collections made in Germany and in Denmark, the number of infected sites was 4 out of 11 for *L. perenne* and 14 out of 20 for *S. pratensis*. The scarcity of isolated wild and semi-wild habitats made collection trips rather inefficient in these countries, especially when the target is meadow fescue.

**Table 1:** *Epichloë* endophyte frequencies in populations of *Schedonorus pratensis, S. arundinaceus, S. giganteus*, and *Lolium perenne* collected in Austria, Denmark, Germany, Italy, Norway, and Sweden (Average infection rates among infected populations; SD, standard deviation; ND, not determined).

Species	Country	No. of collected populations	Infected populations (%)	Average IR (%)	SD (%)	No. of infected plants
*S. pratensis*	Austria	12	100.0	69.3	23.1	149
	Denmark	10	70.0	84.5	23.2	78
	Germany	10	70.0	74.6	10.4	93
	Italy	28	85.7	76.3	24.5	320
	Norway	9	100.0	90.6	10.8	125
	Sweden	42	100.0	87.5	16.3	611
*S. arundinaceus*	Denmark	9	66.7	63.8	37.9	55
*S. giganteus*	Denmark	2	100.0	64.3	20.2	17
	Germany	2	50.0	80.0	ND	12
*L. perenne*	Denmark	7	14.3	8.3	ND	1
	Germany	4	75.0	26.4	23.7	11


A large-scale screening was performed analyzing 255 *S. pratensis* accessions from USDA and 96 from NordGen. Infected accessions were detected with an *in planta* microsatellite-based PCR assay using the loci B10 and B11. The detection threshold of this method, when using bulk samples, was tested with different combinations of *in vitro*–composed admixtures of endophyte infected (E+) and endophyte free (E-) stems. A clear and strong amplification was detected up to bulks with 14 E- stems and 1 E+ stem. This approach allows also to identify the presence of different species or strains in the mixture if they have different amplicon sizes at the chosen loci. Bulks of stems infected with *E. uncinata* and *E. siegelii* were tested and species distinction was possible up to 14:1 ratio. Further ratios were not tested because of the increasing amount of plant material which exceeded the threshold recommended in the DNA extraction protocol. Of the 255 USDA seedlots, 215 were able to grow enough seedlings to be analyzed: 159 were endophyte free, 12 had ambiguous results, and 44 had clear evidence of endophyte presence. Subsequent verification of the 56 (12+44) putative E+ accessions by immunoblotting only confirmed *Epichloë* infection in 12 accessions. The infection rates ranged from 2.5 to 77.5% (mean = 38%; 95% CI ± 16.4%; *SE* = 7.4%; *n* = 12). Regarding the 96 NordGen accessions, only 4 of them did not germinate. Of the remaining, 53 out of 92 (57.6%) were found infected with *Epichloë* endophytes both with the PCR assay and the immunoblot, the infection rates ranged from 3.1 to 100% (mean = 71.2%; 95% CI ± 7.9%; *SE* = 4%; *n* = 52). Twelve accessions had an infection rate of 100% while 4 of them had few weak seedlings that eventually died and could not be used for further analysis. The final outcome was therefore 49 E+ accessions, for a total of 1256 plants.

### Ploidy Level

Two plants per each infected population/accession were analyzed with a flow cytometer to confirm the host ploidy. All the *S. pratensis* collected had 14 chromosomes, thus no *S. pratensis* subsp. *apennina* (2N = 4× = 28 chromosomes) was collected in the Alpine regions. *S. arundinaceus* ecotypes sampled in Denmark were hexaploid with a chromosome number of 2N = 6× = 42, as well as the *S. giganteus* ecotypes collected in Denmark and Germany. All the infected accessions from NordGen and USDA were confirmed to be *S. pratensis* with 2N = 4× = 14 chromosomes except for 3 USDA accessions, collected in Morocco (PI347571, PI347572, and PI347573), which were tetraploid, with a chromosomes number estimated to be 2N = 4× = 28, and a clearly different phenotype from typical meadow fescue. After detailed morphological analysis these accessions were identified as *S. arundinaceus* var. *glaucescens*.

### Characterization of *S. arundinaceus* var. Glaucescens Endophyte

**FIGURE 1:**
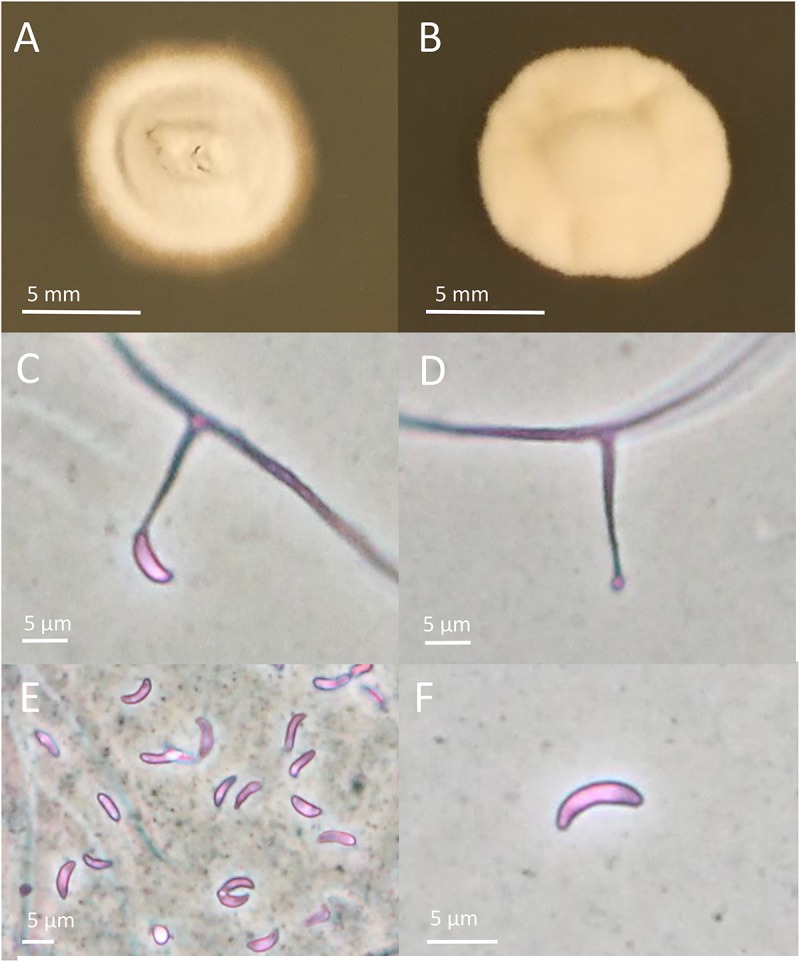
Colony and conidial morphology of *Epichloë* isolates from *Schedonorus arundinaceus* var. *glaucescens*. **(A**,**B)** Different colony morphologies of FaTG-5 isolates. **(C**,**D)** Conidiophores from the two isolates. **(E**,**F)** Conidia from the two isolates. Pictures of colonies were taken after growing on PDA for 4 weeks.

**FIGURE 2:**
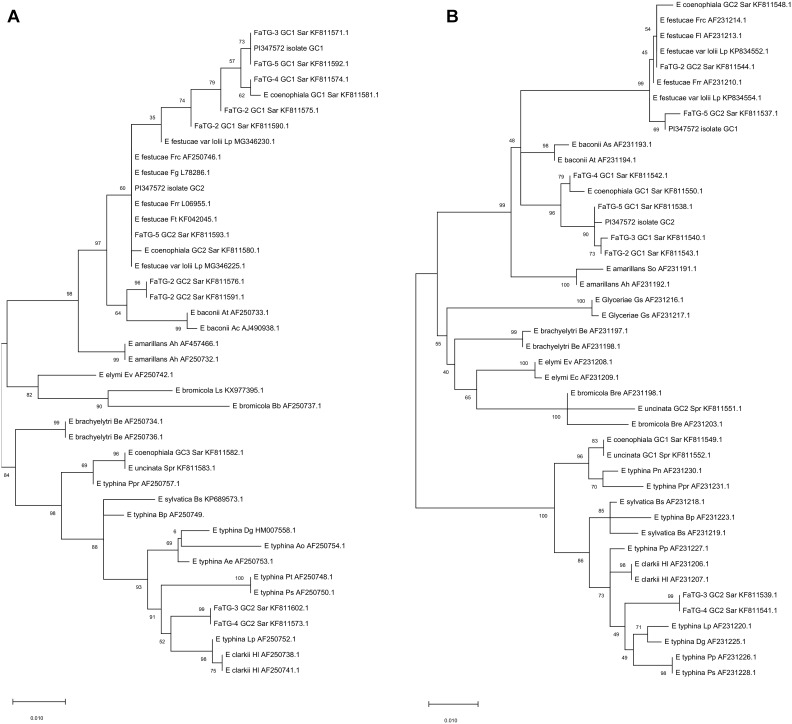
Phylograms derived from maximum likelihood (ML) analysis and Tamura-Nei model of the partial sequence of tubB **(A)** and tefA **(B)** genes from representative haploid *Epichloë* species and hybrid species included in this study. The two copies obtained from *S. arundinaceus* var. *glaucescens* are labeled as “PI347572 isolate.” The tree is midpoint rooted and drawn to scale, with branch lengths measured in the number of substitutions per site. Bootstrap values (1000 replicates) are shown next to the branches. GC stands for gene copy. GenBank accession numbers are provided for each sequence. Letters after each endophyte refer to host designations as follows: Lp, *Lolium perenne*; Dg, *Dactylis glomerata*; Ps, *Poa sylvicola*; Pp, *Phleum pratense*; Sar, *Schedonorus arundinaceus*; HI, *Holcus lanatus*; Bp, *Brachypodium pinnatum*; Bs, *Brachypodium sylvaticum*; Spr, *Schedonorus pratensis*; Pn, *Poa nemoralis*; Ppr, *Poa pratensis*; Gs, *Glyceria striata*; Be, *Brachyelytrum erectum*; Ev, *Elymus virginicus*; Ec, *Elymus canadensis*; Bre, *Bromus erectus*; So, *Sphenopholis obtusata*; Ah, *Agrostis hiemalis*; As, *Agrostis stolonifera*; At, *Agrostis tennuis*; Frr, *Festuca rubra* subsp. *rubra*; Fl, *Festuca longifolia*; Frc, *Festuca rubra* subsp. *commutata*; Fg, *Schedonorus giganteus;* Bb, *Bromus benekenii*; Ls, *Leymus secalinus*; Pt, *Poa trivialis*; Ao, *Anthoxanthum odoratum.*

The endophytes harbored in PI347571 and PI347573 were formerly classified as FaTG-2 and FaTG-5, respectively (Ekanayake et al., 2012), therefore isolates from the accession PI347572 that have not been examined before were cultured on PDA and characterized (Figure 1). The diameters of the colonies after 4 weeks at 24°C were 10–15 mm, the mycelium was white, densely velvety, slightly raised in the center, more flattened toward the perimeter, ca. 1 mm submerged at the margin, whereas the reverse side of the colonies were light brown with some fractures of the agar medium. Sporulation was moderately abundant. Conidiogenous cells were 10–25 μm long, ca. 2 μm wide at the base, tapering to ca 0.5 μm at the apex, arising solitarily from hyphae, and usually lacking basal septum. The conidia (6.4–8.5 μm × 1.8–3.5 μm) were 6–10 μm long, 1–3 μm wide, luniform to reniform, hyaline, aseptate, and smooth. The presence of two amplicons at B10 and B11 suggested that the isolates were interspecific hybrids, this was confirmed by the phylogenetic analysis of the *tef*A and *tub*B (Figure 2) genes. Both genes were present in two copies. In the *tefA* based phylogeny, copy 1 (GC1) was placed in a clade with FaTG-5 among *E. festucae* and a *E. festucae* var. *lolii* strains. Gene copy 2 (GC2) formed a separate subclade with all the other *S. arundinaceus* isolates (*E. coenophiala*, FaTG-2, FaTG-3, FaTG-4, and FaTG-5) nested within a larger clade including *E. baconii* and *E. amarillans* reference strains. In the *tub*B phylogram the GC1 formed again a separate subclade with all the other *S. arundinaceus* taxonomic groups next to *E. festucae* var. *lolii.* that seemed to be genetically close. The GC2 of *tub*B showed genetic similarities with those of FaTG-5 and *E. festucae.* By contrast, FaTG-2 strains in the *tub*B tree were more similar to *E. baconii* and FaTG-3/FaTG-4 more similar to *E. typhina.* Moreover, SSR profiles of the endophyte from PI347573 that has been previously identified as FaTG-5 showed close similarity with the isolate from PI347572. Taken together, evidence suggest that the newly characterized endophyte from *S. arundinaceus* var. *glaucescens* is correctly assigned to FaTG-5.

### SSR and Population Genetic Analysis

The allelic diversity of 250 isolates (224 *E. uncinata*, 9 *E. coenophiala*, 7 *E.* festucae, 6 *E. festucae* var. *lolii*, 1 *E. siegelii*, 1 FaTG-2, and 2 FaTG-5) was investigated using 7 microsatellites. The isolates were a representative subset of the screened populations. A detailed list of the samples and their allelic profiles at each locus is provided in Supplementary Table S2, Supporting information. All seven microsatellites yielded amplicons in the above mentioned species except for E08 in *E. festucae* and *E. festucae* var. *lolii*. The number of alleles at the 7 loci spanned from 0 to 5, according to the endophyte species. Also, the information index at each locus varied among species (Table 2): B11 and E33 were the most polymorphic loci in *E. festucae* var. *lolii* and *E. festucae*, whereas for *E. uncinata* there was higher variability at B10. It was impossible to obtain the same information for *E. siegelii*, FaTG-2 and FaTG-5 because only one haplotype was available, and for *E. coenophiala*, because the software GenAlex cannot process triploid data.

**Table 2:** Number of alleles, number of effective alleles, and informativeness at the seven SSR loci analyzed for each endophyte species.

Pop	Locus	*N*	Na	Ne	*I*
*E. festucae*	B10	7	2	1.324	0.410
	B11	7	3	2.333	0.956
	E33	7	3	2.333	0.956
	E36	7	1	1.000	0.000
	E29	7	3	1.815	0.796
	E39	7	1	1.000	0.000
	E08	0	0	0.000	0.000
*E. festucae* var. *lolii*	B10	8	2	1.280	0.377
	B11	8	5	3.200	1.386
	E33	8	2	1.280	0.377
	E36	8	1	1.000	0.000
	E29	8	3	1.684	0.736
	E39	8	3	1.471	0.602
	E08	0	0	0.000	0.000
*E. uncinata*	B10	224	4	3.838	1.365
	B11	224	3	1.018	0.057
	E33	224	3	2.657	1.037
	E36	224	3	2.089	0.784
	E29	224	2	2.000	0.693
	E39	224	2	1.577	0.552
	E08	224	3	2.623	1.027


*N, sample size; Na, number of alleles; Ne, number of effective alleles; I, Shannon’s information index*.

**FIGURE 3:**
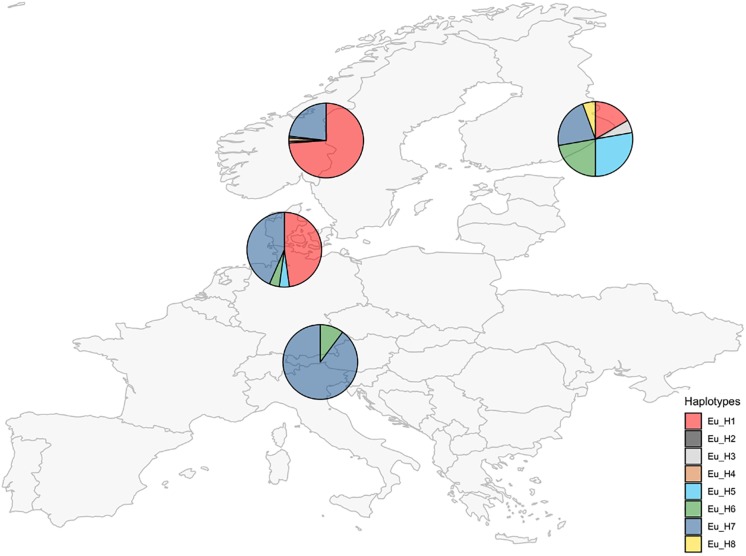
Occurrence of *E. uncinata* haplotypes (defined in Table 3) in four macro areas.

Unique combinations of alleles across the 7 different loci were called different haplotypes (Table 3). A total of 21 haplotypes were identified among the 250 isolates. The expected number of alleles was detected in 97% of cases and it was consistent with the information currently available on the closest extant relatives or the ancestral species when the sample was a hybrid. The locus E08 was not detected in the *E. festucae* strains analyzed, accordingly, all the hybrids that have *E. festucae* among their ancestors (*E. siegelii*, FaTG-2, FaTG-5, and *E. coenophiala*) lacked this allele. B11 was not detected in *E. typhina* (Moon et al., 1999) therefore only one amplicon was detected in its derived hybrids *E. uncinata* and *E. coenophiala*. Fewer alleles than expected were only observed at B10 in *E. siegelii*, at E33 in FaTG-2 and at E39 in three haplotypes of *E. uncinata* (Eu_H1, Eu_H2, and Eu_H4 where one amplification product instead of two was detected). *E. coenophiala* had 3 haplotypes which were characterized by three alleles at loci B10, E33, E36, and E39 and two at the remaining ones. The most abundant tall fescue haplotype collected in Zealand (Denmark) was Ec_H1, isolated in 5 different locations. Ec_H2 was isolated from two samples collected in a relatively small area around Stevns Klint (Stevns Municipality, Zealand, Denmark) and it can be distinguished from the previous haplotypes for a different amplicon size at B11 (150–179 bp vs. 150–194 bp, respectively). In comparison, isolates from Spain had a different profile at B10, B11, and E29. The most abundant *E. festucae* haplotype found in this study was Ef_H1 isolated both in Denmark and in Germany. Ef_H1 is very similar to Ef_H2 and Ef_H3, from which it differs at only one locus, but very different from Ef_H4 with differences at 4 loci. Interestingly, Ef_H4 was isolated from a single location in Fyn (Denmark) where the majority of the plants collected nearby were infected with Ef_H3. Isolates from *E. festucae* var. *lolii* collected in Denmark and Germany had the same genetic profile, named El_H1, and differed from isolates used as internal standard, collected in Spain (El_H2) and in Italy (El_H3), at one locus. *E. uncinata* showed an unbalanced distribution of the 8 detected haplotypes. The 224 *E. uncinata* isolates were grouped in 4 populations (Figure 3) according to the macro area of their geographic origins (Supplementary Table S2). Samples collected in Italy, Austria, and southern Germany were grouped in the population *Alps*, samples from Denmark and northern Germany form the population *North-West*, samples from Norway and Sweden were grouped in the population *Scandinavia* and the remaining samples from Finland, Russia, and Kyrgyzstan were grouped in the population *East*. The most frequent haplotypes were Eu_H1 (113 samples) and Eu_H7 (88 samples). Eu_H1 is the most abundant haplotype in *Scandinavia*, found in 73.9% of the isolates, represents 47.8% of the isolates from the population *North-West* and 16.7% of the isolates from *East*, but is completely absent in the *Alps*. Eu_H7 is the only haplotype shared among the four populations, it is the most abundant in the *Alps* (87.8%), it is as abundant as Eu_H1 in the population *North-West* (43.5%), and present with lower percentages in *Scandinavia* (23.1%) and *East* (22.2%). Eu_H2 and Eu_H3 were the only two haplotypes with a polymorphism at B11, they were isolated from a single population, respectively in *Scandinavia* and *East*. Eu_H4 was only found in two locations in *Scandinavia*. Eu_H5 was the most abundant haplotype in *East* (27.8%), isolated in a single population both in *Scandinavia* and *North-West*, but completely absent in the *Alps*. Eu_H6 was the only other haplotype isolated on the *Alps* (12.2%), fairly abundant in *East* (22.2%), present in only one isolate in *North-West* but completely absent in *Scandinavia*. Eu_H8 was only found in a single population in *East*. During the screening, genotypes from 47 locations were found infected with more than one haplotype. Among the Scandinavian collected samples, when two haplotypes were detected in the same location, one was Eu_H1 and the other was always Eu_H7, except for one case when a Eu_H4 was found. In the *Alps*, the second haplotype in addition to the common Eu_H7 was always Eu_H6.

**Table 3:** Haplotypes of *Epichloë* isolates based on microsatellite profiles of seven loci.

				Alleles size (bp)								
Host	Endophyte	Haplotype	No. of isolates	B10	B11	E33	E36	E29	E39	E08	Country	Closest non-hybrid groups
Sa	*E. coenophiala*	Ec_H1	5	164 173 188	150 194	326 329 344	398 407 414	122 150	419 424 426	211 238	DK	Efe, ETC, LAE
		Ec_H2	2	164 173 188	150 179	326 329 344	398 407 414	122 150	419 424 426	211 238	DK	Efe, ETC, LAE
		Ec_H3	2	175 181 185	125 194	326 329 344	398 407 414	122 134	419 424 426	211 238	ES	Efe, ETC, LAE
											
Sag	FaTG-2	Fa_H1	1	178 181	131 150	344	398 405	134 137	424 430	245	MO	Efe, LAE
Sag	FaTG-5	Fa_H2	2	176 190	131 150	344 349	398 405	134 150	424 430	245	MO	Efe, LAE
											
Sg	*E. festucae*	Ef_H1	4	181	150	344	398	137	419	–	DK, DE	Efe
		Ef_H2	1	181	119	341	398	134	419	–	DE	Efe
		Ef_H3	1	181	150	341	398	137	419	–	DK	Efe
		Ef_H4	1	187	154	348	398	130	419	–	DK	Efe
												
Lp	*E. festucae* var. *lolii*	El_H1	4	181	179	344	398	134	430	-	DK, DE	Efe
		El_H2	1	181	160	344	398	134	430	-	ES	Efe
		El_H3	1	181	209	344	398	131	430	-	IT	Efe
												
Sp	*E. siegelii*	Es_H1	1	188	117 125	335 346	398 410	122 140	430 434	202	DE	Efe, Ebr
												
Sp	*E. uncinata*	Eu_H1	113	164 200	121	326 335	407 410	122 146	426	209 228	DK, DE, SE, NO, FI, RU	Ebr, ETC
		Eu_H2	1	164 200	117	326 335	407 410	122 146	426	209 228	SE	Ebr, ETC
		Eu_H3	1	164 200	109	326 333	407 410	122 146	421 426	209 228	RU	Ebr, ETC
		Eu_H4	2	164 200	121	326 335	398 407	122 146	426	209 228	SE	Ebr, ETC
		Eu_H5	7	164 200	121	326 335	398 407	122 146	421 426	209 228	DE, FI, KZ, RU, SE	Ebr, ETC
		Eu_H6	10	164 200	121	326 333	407 410	122 146	421 426	209 228	DE, IT, RU	Ebr, ETC
		Eu_H7	89	176 196	121	326 333	407 410	122 146	421 426	206 228	AT, DE, DK, IT, KZ, NO, RU, SE	Ebr, ETC
		Eu_H8	1	176 196	121	326 335	398 407	122 146	421 426	206 228	RU	Ebr, ETC


*Sa, *S. arundinaceus*; Sag, *S. arundinaceus* var. *glaucescens;* Sg, *S. giganteus*; Lp, *L. perenne*; Sp, *S.* pratensis; Efe, *E. festucae;* ETC, *E. typhina* complex; LAE, *Lolium-*associated clade; Ebr, E. bromicola.*

The number of multi-locus genotypes (MLG) detected span from 2 to 6 and incidence was independent of the population size (*R*^2^ = 0.0034). Genotypic diversity indices (Table 4) displayed consistently low diversity in all populations: Shannon-Weiner’s index (H) ranged from 0.37 (*Alps*) to 1.64 (*East*; Stoddart and Taylor’s corrected index (G′) ranged from 1.25% (*Scandinavia*) to 26.44% (*East*; and Simpson’s corrected index (λ′) ranged from 0.22 (*Alps*) to 0.84 (*East*). Therefore *East* was clearly the population with the highest diversity and Alps was the population with the lowest diversity according to all indexes except for G′, but this is due to the different sensitiveness of the index to changes in abundant genotypes or in rare alleles. Overall, the populations were very similar to each other as shown by the pairwise comparisons (Table 4) based on Nei’s unbiased genetic identity, where all values where close to 1, and on Nei’s unbiased genetic distance, where all values were close to 0. The two populations that differed the most are the *Scandinavia* and the *Alps*. Evenness (E.5) ranged from 0.61 (*Alps*) to 0.90 (*East*) showing a relatively unbalanced distribution of haplotypes in the *Alps*.

**Table 4:** Statistics summarizing genotypic richness, diversity, and evenness in 224 *E. uncinata* isolates collected in 4 macro areas: population name as defined in Supplementary Table S1 (Pop), number of multi-locus genotypes observed (MLG), Shannon-Wiener Index of MLG diversity (H), Stoddart and Taylor’s Index scaled by sample size (G’), Simpson’s index corrected for sample size (λ’), and Eveness (E.5).

Pop	N	MLG	H	G′ (%)	λ′	E.5	Alps	North-West	East	Scandinavia
Alps	49	2	0.37	2.59	0.22	0.61	–	0.95	0.94	0.88
North-West	23	4	0.99	10.30	0.60	0.82	0.05	–	1.00	1.00
East	18	6	1.64	26.44	0.84	0.90	0.06	0.00	–	0.98
Scandinavia	134	5	0.70	1.25	0.40	0.66	0.13	0.00	0.02	–


*On the right, pairwise comparison of the four populations based on Nei’s unbiased genetic identity (above diagonal) and Nei’s unbiased genetic distance (below diagonal) calculated in GenAlEx V6.5 (Peakall and Smouse, 2012).*

### Population Structure

**FIGURE 4:**
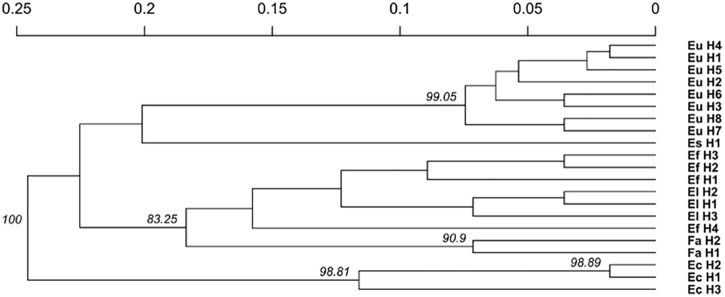
Unweighted pair group mean average (UPGMA) cladogram with 10000 bootstrap replicates based on a microsatellite profile-based distance matrix, genetic bootstrap values greater than 75% are shown. The labels refers to the name of the haplotypes described in Table 3.

The relationship between isolates was investigated using the Provesti’s distance inferred from clone-corrected microsatellite data. The phenogram (Figure 4) generated with the UPGMA method, resolved 3 main clusters that split into sub clusters that were consistent with the host species from which the haplotypes were isolated. Cluster 1 consisted of the two *S. pratensis* endophytes, separated in two clades. The first clade included the eight *E. uncinata* haplotypes. Eu_H1, Eu_H2, Eu_H4, and Eu_H5 cluster together because they only differs at one or two loci, Eu_H3 and Eu_H6 are closely related because they have the same profile at E33 and E39, suggesting that the first might derive from the other through modification at locus B11; the haplotypes Eu_H7 and Eu_H8 group separately from the others having amplicons of different size at B10 and E29. The second clade of cluster 1 includes the only *E. siegelii* isolate currently known. Cluster 2 included *E. festucae, E. festucae* var. *lolii* and the newly characterized isolates from *S. arundinaceus* var. *glaucescens*. Although FaTG-2 and FaTG-5 haplotypes were in a distinct clade, *E. festucae* and *E. festucae* var. *lolii* showed to be closely related, as expected, but still different enough to group in two different subclades. The only exception is haplotype Ef_H4 that appeared to be different from the other *E. festucae* and *E. festucae* var. *lolii* isolates and clustered separately from them. Cluster 3 included *E. coenophiala*, its allotriploid-like genome makes it very distinct from the others. The phenogram was supported by Mantel test statistics with the original and derived matrices showing a high cophenetic correlation (*r* = 0.992).

**FIGURE 5:**
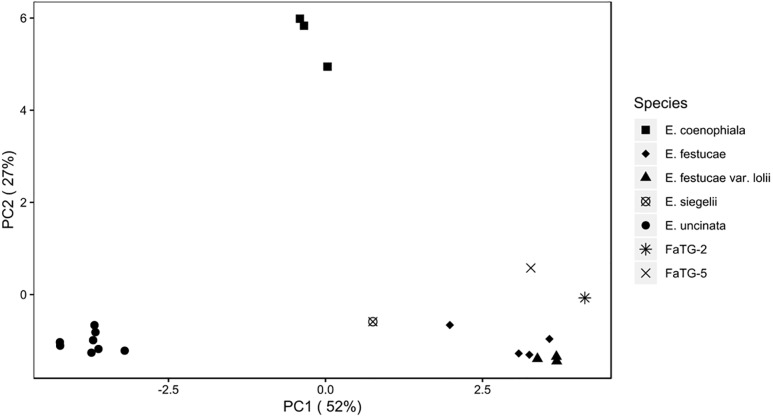
Analysis of principal components (PCoA) scatterplot of the *Epichloë* haplotypes.

**FIGURE 6:**
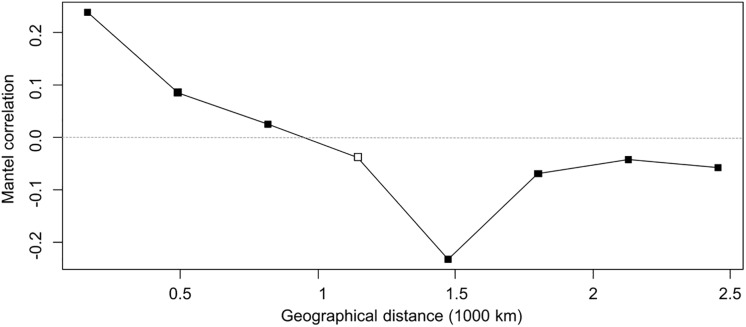
Mantel correlogram. Filled squares indicate significant positive or negative correlations (based on sequential Bonferroni corrections with α = 0.05) between genetic and geographical distance of *E. uncinata* isolates. Open squares indicate non-significant correlation.

Similarly, the PCoA (Figure 5) showed varying degrees of population separation according to principal component PC1 and PC2, which explained 52 and 27% of the variance, respectively. The 7 species clustered consistently with their species and genetic composition on the *x*-axis and with their ploidy on the *y*-axis: on the left side *E. uncinata* haplotypes, whose ancestors are *E. bromicola* and *E. typhina*, group together; on the right side there are the *E. festucae* strictly related species, whereas at the center of the plot there are *E. siegelii*, whose ancestors are *E. festucae* and *E. bromicola*, and, in the upper part, the allotriploid-like *E. coenophiala* which share the same *E. typhina* ancestor as *E. uncinata*, the same LAE ancestor as FaTG-2 and FaTG-5. The Mantel correlation between pairwise Provesti’s genetic distance and pairwise geographic distances (measured in kilometers) between populations was equal to 0.226 (*p* < 0.0001). Thus, populations close to each other tend to be genetically more similar than expected by chance, and genetic differences increase with geographic distances. In order to study the relationship between genetic and geographic distances across space and its variations in the correlations a Mantel correlogram (Figure 6) was computed on 216 *E. uncinata* samples whose coordinates were available.

The correlogram, with five distance classes, showed an overall significance since 7 out of 8 correlation coefficients were significant. Populations distant by 164 km tend to be similar (*r* = 0.24; *p* < 0.0001 with 9999 permutations), this indicates that the haplotypes composition is more similar than they would by chance at the shortest distances. Mantel correlation decreased almost linearly up to -0.23 (*p* < 0.0001) for populations distant approximately 1500 km from each other. From 1800 km onward the correlation tends to stabilize on an average level of about -0.06 (*p* < 0.005) which can be related to patches of genetic variation such as those areas in Sweden and *East* where some atypical haplotypes (Eu_H2-3-4-8) were isolated. A negative correlation indicates more dissimilar haplotypes than expected by chance on farther distances. Mantel correlations were however, not high, therefore the spatial structure is not strong, and only 5% of the genetic divergence is explained by geographic distance.

### Loline Analysis

**FIGURE 7:**
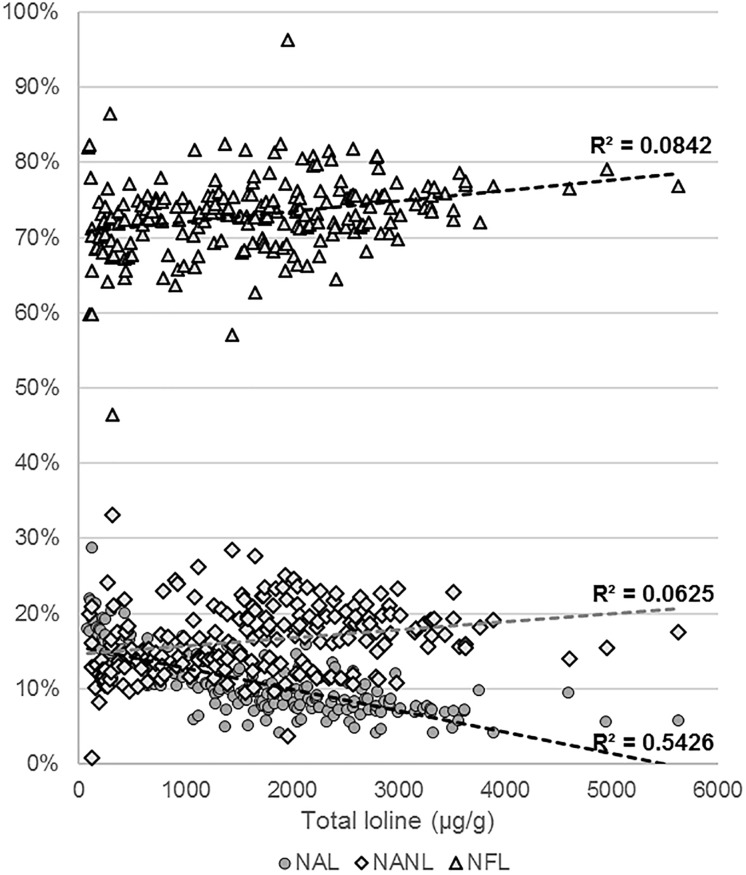
Variation of the NAL, NANL, and NFL proportions with the increase of the total amount of loline.

Levels of NAL, NANL, and NFL were measured in 218 samples of endophyte-infected S. pratensis (Supplementary Table S3). The total concentration of loline, calculated as sum of the concentrations of the single compounds, varied widely among isolates, spanning from barely detectable traces (<25 μg/g) up to 5629 μg/g. The average profile composition of the isolates was NAL = 10.9% (95% CI ± 0.6%; *SE* = 3%); NANL = 16.2% (95% CI ± 0.6%; *SE* = 3%); and NFL = 73% (95% CI ± 0.7%; *SE* = 3%). These proportions changed with the increase of the total amount of loline with a calculated *P* < 0.005 (Figure 7): in the range 100–1000 μg/g the average percentage of NANL was 14% (95% CI ± 1.3%; *SE* = 0.7%; *n* = 67) and NFL was 70.9% (95% CI ± 1.4%; *SE* = 0.7%; *n* = 67), but in the range 3000–4000 μg/g they increased to 18.1% (95% CI ± 1%; *SE* = 0.5%; *n* = 17) and 75.3% (95% CI ± 1%; *SE* = 0.5%; *n* = 17), respectively. By contrast, in the same ranges, the average percentage of NAL decreased from 15.2% (95% CI ± 0.8%; *SE* = 0.4%; *n* = 67) to 6.7% (95% CI ± 0.7%; *SE* = 0.3%; *n* = 17). Data suggested that the production of high levels of loline is correlated with a slight increase of the proportion of NANL and NFL (*R*^2^ < 0.1) and with a greater decrease of NAL (*R*^2^ < 0.54).

**FIGURE 8:**
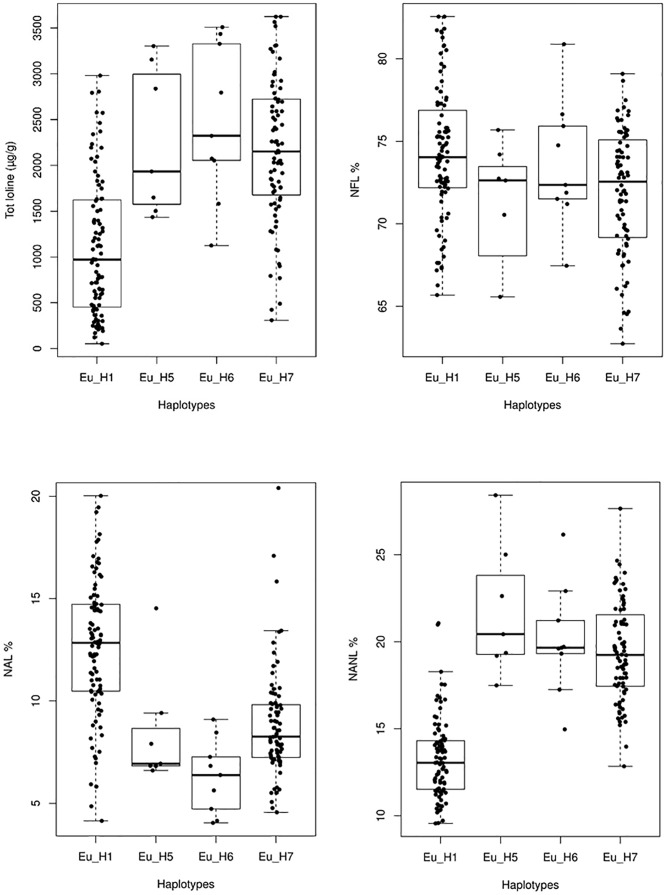
Variation of the total amount of loline and NAL, NANL, and NFL proportions in 4 different haplotypes. Outlier values are not shown.

Significant differences in the average amount of total loline and in the proportion of the loline alkaloids were also found among haplotypes (Figure 8). Specifically, Eu_H1 had a lower amount of total loline (*P* < 0.005) and percentage of NANL (*P* << 0.001) but a higher percentage of NAL (*P* < 0.005) compared to Eu_H5, Eu_H6 and Eu_H7. As it concerns the percentage of NFL, significant differences were found only between Eu_H1 and Eu_H7 (*P* = 0.01).

Traces of NFL (30 μg/g) were detected in the FaTG-2 sample isolated from the accessions PI 347571, but no loline alkaloids were detected in the FaTG-5 isolates.

## Discussion

### Occurrence of Epichloë Endophytes in Wild and Semi-Wild European Meadows

*Epichloë* endophytes are a valuable resource, useful to bring new resistances in forage and turf grasses. Their use in agriculture is well-established and breeding companies are looking for new strains with improved characteristics to introduce in their varieties. Animal-friendly *Epichloë* endophytes are rare and substantial sampling effort is required to identify genotypes with an appropriate alkaloid profile. In this study we screened wild- and semi-wild grass ecotypes collected in different countries or provided by seed banks, after establishing a nursery of *Epichloë* infected plants.

The frequency and the occurrence of *Epichloë* endophytes in Europe is consistent with the results of previous screening (Oliveira and Castro, 1997; Saikkonen et al., 2000; Jensen and Roulund, 2004; Jensen et al., 2007; Zurek et al., 2012). Among all the countries, the highest infection rates were scored in southern Sweden where all sites were found to be infected with *Epichloë* endophytes. A similar incidence was found in Norway although, due to more intensive agriculture in areas where collection was conducted, it was much harder to find isolated areas colonized with semi-wild meadow fescue. The lowest number of infected locations was scored in Denmark, probably due to the lack of isolated habitats and the intensive use of E- forage cultivars which may successfully invade uncultivated areas. Most of the collected perennial ryegrass plants were, indeed, endophyte-free. Surprisingly, the Alps at the Austrian-Italian border was a good source of infected material, although most of the pastures were supposedly cultivated, or derived from cultivated fields. It seems that the use of permanent pastures led to a complex genetic relationship between naturalized and cultivated meadow fescue in these regions, as it has been found for Scandinavian meadow fescue (Fjellheim et al., 2009).

*Epichloë uncinata* and *Epichloë coenophiala* are described to be vertically transmitted within their host, therefore infected meadow and tall fescue plants likely arise from infected seeds. Several studies, mostly focused on tall fescue and perennial ryegrass, stress the selective advantage of infected grasses over endophyte free grasses correlating high infection rates with better performances to water-supply deficit (West et al., 1993; Lewis et al., 1997), increased photosynthetic rate (Belesky et al., 1987) and resistance to insects, nematodes (Elmi et al., 2000), and seed predators (Madej and Clay, 1991). It is known that E+ grasses can have a higher competitive ability and, because of that, infection frequencies should rise over time (Cunningham et al., 1993) especially if strong biotic and abiotic stresses impose selective pressure on E- individuals (Bouton et al., 1993).

It has to be taken into account that all the infection rate values might be biased by the relatively low number of plants (15–20) sampled in each location and by the small sampling area (on average 1000–2000 m^2^), which increases the probabilities that the sampled plants were siblings, although efforts were made to collect plants as widespread as possible. Infection rates lower than 100% occur when E+ and E- plants coexist in the same population. This could happen if the population is in a transition period toward complete infection or, on the opposite side, toward a loss of infection. Another possible explanation is the occurrence of imperfect vertical transmission to seeds (Ravel et al., 1997) or the loss of the endophyte viability in the seeds, which generates E- plants. Moreover, several commercially available varieties are infected with *Epichloë* endophytes although most of them are endophyte free (Holder et al., 1994; Saikkonen et al., 2000), and their extensive use might have affected the species composition and the ratio between infected and non-infected grasses in the area of sampling. According to our results in Sweden, Norway and on the Alps it is highly unlikely to find wild extensive habitats containing *Epichloë*-free meadow fescues.

### Occurrence of *E. uncinata* in *S. pratensis* Accessions

With regard to the accessions from the germplasm banks, the number of infected USDA accessions was surprisingly low, especially according to the immunoblot test, which only confirmed 12 out of 44 PCR-positives. The reason for the discrepancy between the two assays could be that the infection rates of some seedlots were very low due to the loss of viability of the endophytes during the storage. Another possible explanation is that the immunoblot may not have been able to detect the endophyte in the seedlings because of low biomass. Seven accessions were already known to be endophyte infected from previous studies, but when tested with the immunoblot they were negative. Moreover, Holder et al. (1994) tested seeds of 198 meadow fescue accessions, only 30 of them were found to be E+ and they experienced the same phenomenon when assessing the infection status of the seedlings, realizing that infection rates were lower than the ones in the seeds. The loss of endophyte viability is a known problem related to seeds storage conditions which are optimized to preserve seeds’ germination rate rather than endophytes’ viability (Clement et al., 2008). Plants were also severely weakened by a severe powdery mildew (*Blumeria graminis*) infestation and by a long and cold winter. Long exposure to sub-optimal temperatures can strongly affect the amount of endophyte mycelia in the plant, as described by Breen (1992) who demonstrated a difference in the concentration of endophyte in plants grown at constant temperature of 7 or 28°C than in plants growing at a constant temperature of 14 or 21°C. Bacon and Siegel (1988) described the disintegration of the mycelium in leaf sheaths during periods of plant stress and fungal dormancy, which can also justify the failure in detecting the endophytes with the immunoblot method. This is supported by the observation that PCR and immunoblot results concurred for the NordGen accessions, which were assayed during the summer on healthy and vigorous plants. The accessions from NordGen confirmed the trend seen from the collection trips according to which meadow fescue ecotypes in Scandinavian countries, particularly Sweden, were highly infected with *Epichloë* endophytes. For this investigation, only accessions labeled as “wild” or “semi wild” were chosen, but more endophytes can be found also in commercial varieties (Holder et al., 1994). In the NordGen database there are no records of endophyte infected accessions, therefore endophyte infection data have been shared with both genebanks so that they can be incorporated into their databases to enable endophyte and grass scientists to screen for desirable plant-endophyte combinations.

### Identification of FaTG-2 and FaTG-5 Isolates

Identification of endophyte species through morphological traits and host specificity (Christensen et al., 1993) can be difficult and misleading due to similarities among species and intra-species variations of different strains. Most of the current knowledge on taxonomy and phylogenetic relationship among the genus *Epichloë* relies on analysis of intron sequences from the β-tubulin (*tub*B), translation elongation factor 1-α (*tef*A, former *tef*1) and γ-actin (*act*A) (Schardl et al., 1991). Another rapid, cheap, and reliable method to detect and characterize isolates is through microsatellite markers (Moon et al., 1999). Microsatellite fingerprints can be done both in pure cultures and *in planta*. Primers used in this study were specific for endophytes and no amplifications in E- samples were detected. Generally, primers designed for a specific locus work for more than one species and may be used to discriminate isolates not only on a species level but also between taxa and strains (van Zijll de Jong et al., 2003). The level of polymorphisms at each locus, and therefore its informativeness, may be different and change from species to species (Table 2) so that some loci are more suitable to investigate genetic differences in a species than others. The result shown in this study may help to choose an adequate set of loci and help the identification of new isolates by comparison of their genetic profile with the haplotypes described.

In order to correctly identify isolates of the accession PI347572, all three approaches were applied (morphological traits, microsatellite profiles and housekeeping genes phylogeny). Conidia dimensions are consistent with the ones described by Christensen et al. (1993), suggesting that both FaTG-2, and FaTG-5 are characterized by similar shorter conidia (5–8 μm) compared to the ones of *E. coenophiala* (6–15 μm). The microsatellite analysis was able to distinguish the two closely related taxonomic groups, their genetic profiles at B10 and B11 can be compared to the result of other studies (Moon et al., 2004; Jensen et al., 2007) where the strain Tf15, with isozyme phenotype FaC, seems to be closer to FaTG-2, whereas the strain Tf13, with isozyme phenotype FaA, seems to be closer to FaTG-5. Phylogenies inferred from housekeeping genes showed a close relationship of FaTG-2 and FaTG-5 with Lolium-associated endophyte (LAE) clade (Schardl et al., 2008), which seems to be a common ancestor to all the *S. arundinaceus* associated endophytes described so far, and *E. festucae*. This result was consistent with other phylogeny studies based on housekeeping gene sequences (Ekanayake et al., 2013) and SSR-based phenetic analysis (Ekanayake et al., 2012).

### Microsatellite Fingerprinting

Our analysis revealed 21 different haplotypes and amplified a number of amplicons consistent with the one provided in previous studies. Fewer alleles than expected were only observed at B10 in *E. siegelii*, at E33 in FaTG-2 and at E39 in three haplotypes of *E. uncinata*. According to Moon et al. (2004) there could be polymorphisms at the primer sites which prevent a correct ligation of the primer(s) and subsequent amplification of the locus, leading to a false negative; if the amplified fragments, at two or more loci, have the same size they are scored as one allele; a third possibility is the loss of a chromosomal parts.

A direct comparison of the haplotypes is possible at loci B10 and B11 with the results reported in other studies using the same loci (Moon et al., 1999, 2004; Young et al., 2014; Clayton et al., 2017). In *S. arundinaceus*, haplotype Ec_H1 has the same profile as *E. coenophiala* profile 1-1 (Young et al., 2014) at both loci, whereas Ec_H2 has a polymorphism at B11 never described before. Ec_H3 has the same profile as the isolate Tf28 (isozyme phenotype coC) (Moon et al., 1999) and it was only detected from plants collected in Spain. In *S. giganteus, E. festucae* haplotypes Ef_H1 and Ef_H3 have the same profile as the isolate Frc7 (Moon et al., 1999), which was isolated from *Festuca rubra* subsp. *commutata*. Regarding isolates from *L. perenne*: El_H1 has the same profile as strains Lp5, Lp6 and Lp13 (isozyme phenotype loA) (Moon et al., 1999) whereas the other haplotypes are different from the ones previously described. The only isolate of *E. siegelii* had the same profile as ATCC 74483, an attempt to re-isolate *E. siegelii* from the original accession was made but it was unsuccessful. Regarding *S. pratensis* isolates, at locus B11 only one allele with size 121 pb has been described so far in *E. uncinata*, which was the most common found in this study as well, but two isolates had an amplicon of 117 pb (Eu_H2) and 109 (Eu_H3), respectively. These polymorphisms are likely to arise by errors during the replication process, due for instance to DNA polymerase slippage. At B10 the haplotypes split into two groups, one with amplicons of approximately 164 and 200 bp and the other with amplicons of approximately 176 and 196 bp, which reflect the same allele sizes as the two strains isolated from Fp1 and Fp4 described by Moon et al. (1999) with allozymes profile unA and unB, respectively.

Clayton et al. (2017) have described two similar haplotypes naming them “ecotype 1” and “ecotype 3,” and their B10 sequences are compared to the ones of the closest *E. uncinata* ancestors *E. typhina* and *E. bromicola.* There are several variations in repeated structures between the two copies of the locus among the two haplotypes and this raises the question whether these variations occurred in *E. uncinata* or in its ancestors, assuming different independent interspecific hybridization events. If two independent anastomosis events occur in different locations an unbalanced geographical distribution of the haplotypes may be expected since *E. uncinata* is only vertically transmitted. Eu_H1 is the most abundant haplotype in the northern countries and it is completely absent in Italy and Austria, whereas Eu_H7 can be found throughout Europe, even in Russia and Kyrgyzstan. Its wide distribution might be anthropogenic if, for example, this haplotype was infecting one or more cultivars commercialized in several countries. Contamination of natural populations with plants from infected varieties could also explain the presence of two haplotypes in the same location. Clayton et al. (2017) come to a similar conclusion, associating different geographical origins to the 4 ecotypes detected: the strain U2 with a similar profile as Eu_H1 may come from Norway and U4, similar to Eu_H7, from Germany.

It can also be speculated that a possible explanation for the observed genetic variation in relation to the geographical distribution involves local adaptation to specific stresses which may positively select one haplotype over another. This could explain the absence of Eu_H1 on the Alps. Clayton et al. (2017) showed that polymorphisms at B10 can lead to changes in the polypeptide sequences since it lies within the exon of an expressed gene, and the two haplotypes have two different allozyme profiles (unA and unB), which could represent a phenotypic diversity on which selection could act.

The above mentioned uneven distribution of the haplotypes and the fact that 5 of the 8 haplotypes scored came from very diverse USDA accessions, reported to be collected in Russia, affected the relation between geographical distribution and genetic diversity investigated with the Mantel correlogram. Having on the shortest distance individuals which are genetically closer than random individuals is partially coherent with what it is expected from clonally propagated species. But the fact that on the longer distance they are more diverse than random individuals does not fit with the idea of a single strain vertically transmitted throughout Europe, it rather supports the hypothesis of different hybridization events.

It has to be taken into account that the number of haplotypes strongly depends on the number and on the information content of the profiled loci, and the number of individuals and populations sampled. Using a wider panel of SSRs on more samples may lead to a deeper characterization of the isolates. The 7 microsatellites used in this study allowed a clear separation between species as shown in the phylogenetic tree and in the PCoA, but as it is clear from the latter, and genetic variation within isolates of vertically propagated species is usually very low due to the absence of sexual reproduction (van Zijll de Jong et al., 2008). For a deeper characterization and to investigate the genetic variation within asexual species it is suggested to either screen more loci or to use a different fingerprinting approach. The phenetic relationships between haplotypes were consistent with those described in other studies (van Zijll de Jong et al., 2003; Karimi et al., 2012) with *E. festucae* and *E. festucae* var. *lolii* being closely related, *E. coenophiala* clustering in a different clade, FaTG-2 and FaTG-5 being genetically very similar and related to *E. festucae*.

### Loline Analysis

One of the features that makes *E. uncinata* an endophyte appealing for agricultural uses is its production of high level of loline alkaloids. Measuring the alkaloids at a given time is like taking a screenshot on a state that it is known to vary greatly during the host lifespan. There are several factors that can directly affect the concentration of these compounds, which include the season of the year (Patchett et al., 2009), pest feeding on the host or abiotic stresses (Patchett et al., 2008; Helander et al., 2016), or that can indirectly affect the amount of alkaloids that the endophyte can produce by altering the mycelium biomass in the host (Ryan et al., 2015). Nevertheless, comparing the amount of loline alkaloids in plants grown at the same conditions is one way to select suitable candidates for being artificially inoculated in elite varieties and re-tested for loline content in the new host.

The total amount of loline measured in this study is consistent with the results of similar studies based on plants grown in pots at greenhouse conditions (Leuchtmann et al., 2000; Patchett et al., 2011). Usually, plants grown in open fields have higher levels of total loline that can easily exceed 10,000 μg/g (Barker et al., 2015). NFL was found to be the alkaloid with the highest concentration in all the isolates, but the proportion of NANL and NAL changed significantly with the increase of the amount of total loline and among haplotypes (Figure 7, 8). Findings from Eu_H1 support the trend found in the literature previously mentioned, where concentrations of NAL are similar or higher than those of NANL, whereas in Eu_H5, Eu_H6 and Eu_H7 from this study the trend was the opposite way. Haplotypes synthesizing higher levels of loline produce significantly more NANL and less NAL than the ones with low level of total loline. These phenotypic differences may be additional evidence for a greater genetic diversity than previously assumed among haplotypes and may be related to differences in their ancestors.

## Conclusion

The Scandinavian northern regions and the Alps are a good source of *Epichloë* infected meadow fescue. Also, germplasm repositories represent a valuable resource for endophytes even though their viability in the seeds is reduced over time, particularly at suboptimal storage conditions. Results from this study provide valuable information to germplasm banks which will allow to provide additional support to forage and turf breeders. The genetic diversity and allelic composition of asexual, vertically transmitted species appears to be more complex than previously assumed. In this study we discuss the possibility of multiple hybridization events as source of intraspecific variability. It is not possible to infer from our results where the hybridization events took place, because meadow fescue and other grasses have been spread by human beings throughout the continent(s). Since our current knowledge of endophyte distribution is incomplete, population genetic studies on ancestor species from a wide geographic range are needed to understand the evolutionary origin of the hybrid endophytes of the *Festuca-Lolium* complex. Moreover, the different genetic background of the haplotypes may affect the production of loline alkaloids. Finally, sharing of the genetic profiles of screened isolates is crucial for identifications of new isolates that may have improved characteristics and could be used for grass breeding and future research.

## Data Availability

The datasets generated for this study can be found in NCBI: https://www.ncbi.nlm.nih.gov/nuccore/MK423914.1, https://www.ncbi.nlm.nih.gov/nuccore/MK423915.1, https://www.ncbi.nlm.nih.gov/nuccore/MK423916.1, and https://www.ncbi.nlm.nih.gov/nuccore/MK423917.1.

## Author Contributions

GC, NR, CJ, and TA designed the experiment. GC, NR, FF, and AL collected the data. GC and AL analyzed the data. All authors contributed to manuscript preparation, editing, and gave final approval for publication.

## Conflict of Interest Statement

GC, NR, and CJ are employees of DLF Trifolium A/S. The remaining authors declare that the research was conducted in the absence of any commercial or financial relationships that could be construed as a potential conflict of interest.

## Supplementary Material

FIGURE S1 Map of the collection sites of the sampled grass ecotypes (blue) and of the screened accessions from USDA (green) and NordGen (red) whose coordinates were available.

TABLE S1 Ecotypes and accessions screened in this study followed by coordinates, altitude, infection status and infection rate.

TABLE S2 List of fingerprinted isolates and their allelic profiles at 7 microsatellites loci. Information about host, endophyte and location of the collection are provided together with the genetic profile of each sample.

TABLE S3 Levels of N-formylloline (NFL), N-acetylloline (NAL), N-acetylnorloline (NANL) in 218 pseudostems of *Epichloë*-infected grasses.
