# Supplementary figures and images for: Large Scale Screening of Epichloë Endophytes Infecting Schedonorus pratensis and Other Forage Grasses Reveals a Relation Between Microsatellite-Based Haplotypes and Loline Alkaloid Levels
Source: Front Plant Sci. 2019 Jun 12;10:765. doi: 10.3389/fpls.2019.00765 (PMC6582706; doi:10.3389/fpls.2019.00765)

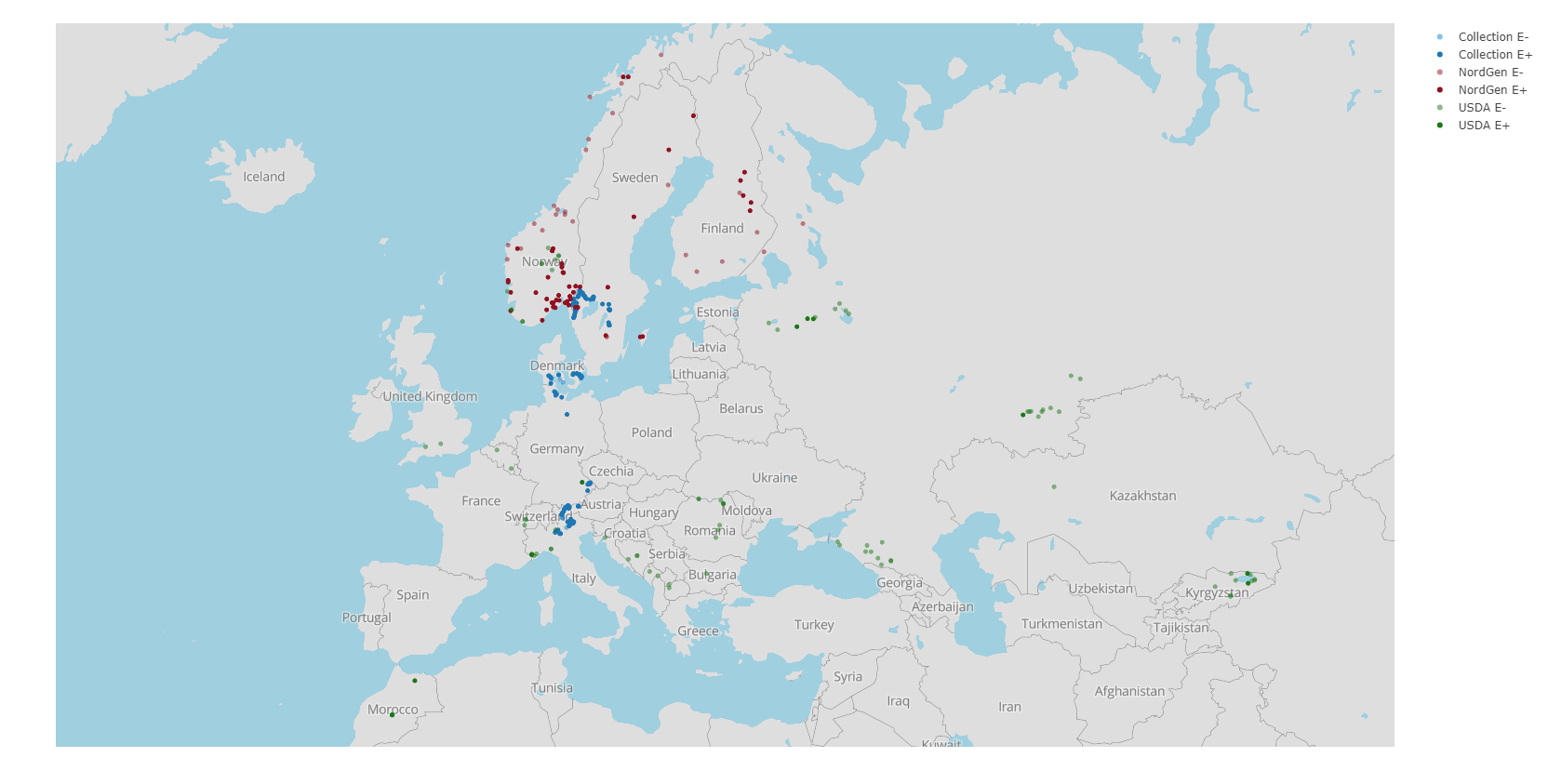

Supplement: FIGURE S1 — Map of the collection sites of the sampled grass ecotypes (blue) and of the screened accessions from USDA (green) and NordGen (red) whose coordinates were available. [file Image_1.PNG]
